# The effectiveness of E-learning in continuing medical education for tuberculosis health workers: a quasi-experiment from China

**DOI:** 10.1186/s40249-021-00855-y

**Published:** 2021-05-18

**Authors:** Zi-Yue Wang, Li-Jie Zhang, Yu-Hong Liu, Wei-Xi Jiang, Jing-Yun Jia, Sheng-Lan Tang, Xiao-Yun Liu

**Affiliations:** 1 China Centre for Health Development Studies, Peking University, Beijing, 100191 China; 2 Beijing Chest Hospital, Capital Medical University, No. 97 Ma Chang, Tongzhou, Beijing, 101149 China; 3 Clinical Centre on Tuberculosis, Chinese Centre for Disease Control and Prevention, No. 97 Ma Chang, Tongzhou, Beijing, 101149 China; 4 Global Health Research Centre, Duke Kunshan University, No. 8 Duke Avenue, Kunshan, 215316 Jiangsu China; 5 School of Mathematical Science, Nankai University, No 94. Weijin Road, Tianjin, 300071 China

**Keywords:** Continuing medical education, Training, Tuberculosis, E-learning, Program evaluation

## Abstract

**Background:**

Given the context of rapid technological change and COIVD-19 pandemics, E-learning may provide a unique opportunity for addressing the challenges in traditional face-to-face continuing medical education (CME). However, the effectiveness of E-learning in CME interventions remains unclear. This study aims to evaluate whether E-learning training program can improve TB health personnel’s knowledge and behaviour in China.

**Methods:**

This study used a convergent mixed method research design to evaluate the impact of E-learning programs for tuberculosis (TB) health workers in terms of knowledge improvement and behaviour change during the China-Gates TB Project (add the time span). Quantitative data was collected by staff surveys (baseline *n* = 555; final *n* = 757) and management information systems to measure the demographic characteristics, training participation, and TB knowledge. Difference-in-difference (DID) and multiple linear regression models were employed to capture the effectiveness of knowledge improvement. Qualitative data was collected by interviews (*n* = 30) and focus group discussions (*n* = 44) with managers, teachers, and learners to explore their learning experience.

**Results:**

Synchronous E-learning improved the knowledge of TB clinicians (average treatment effect, ATE: 7.3 scores/100, *P* = 0.026). Asynchronous E-learning has a significant impact on knowledge among primary care workers (ATE: 10.9/100, *P* < 0.001), but not in clinicians or public health physicians. Traditional face-to-face training has no significant impact on all medical staff. Most of the learners (57.3%) agreed that they could apply what they learned to their practice. Qualitative data revealed that high quality content is the key facilitator of the behaviour change, while of learning content difficulty, relevancy, and hardware constraints are key barriers.

**Conclusions:**

The effectiveness of E-learning in CME varies across different types of training formats, organizational environment, and target audience. Although clinicians and primary care workers improved their knowledge by E-learning activities, public health physicians didn’t benefit from the interventions.

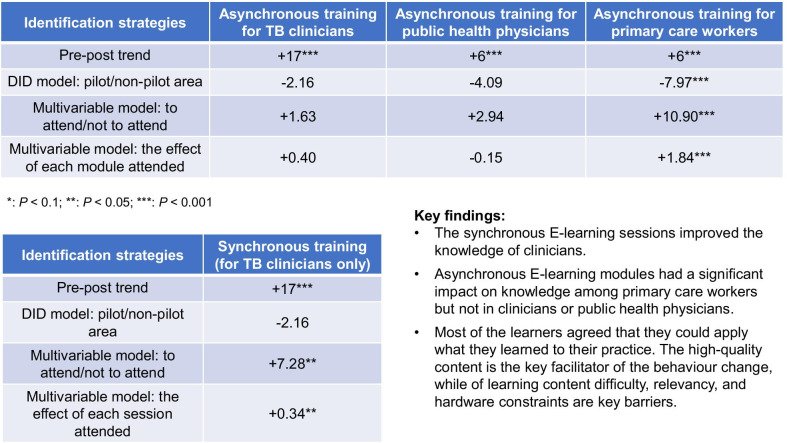

**Supplementary Information:**

The online version contains supplementary material available at 10.1186/s40249-021-00855-y.

## Background

Continuing medical education (CME) is an “established method that can facilitate lifelong learning, which focuses on maintaining or developing knowledge, skills, and relationships to ensure competent practice” [1]. Given the context of rapid technological advancement in medicine, it has been proposed that CME may play a more important role in updating the physicians’ knowledge and skills as well as improving the quality of care. Since the 1980s, considerable resources have been invested in developing various CME programs in China and globally [2, 3]. However, the effectiveness of CME interventions remains unclear [4–8]. Existing evidence has shown that traditional didactic sessions have little or no impact, while interactive or mixed workshops were more likely to associated with some positive effects, as revealed by two systematic reviews [1, 6]. In addition, the clinicians are usually too busy in their daily practices to attend training. Lack of high-quality learning resources, unattractive and irrelevant training contents were also key barriers to their learning [9]. Therefore, in order to improve the performance of physicians and the health system, it’s important to find an appropriate CME training format that could increase access to high-quality training resources, considering health workers’ needs in terms of content, timing, and location.

E-learning provides a unique opportunity for addressing these challenges in CME. Due to the COVID-19 pandemic, we saw an unprecedented explosion of online and remote training in 2020 [10, 11]. Existing evidence indicated that E-learning could reduce the cost [12, 13], improve the access to education [14], as well as provide more flexibility for students who have work and family commitments [15, 16]. However, whether E-learning can improve student outcomes remains controversial [16–18]. In addition, most of the available evidence comes from higher education-based studies [19–21]. Little E-learning research was conducted in CME settings [20, 22, 23], especially in low- and middle-income countries (LMICs) [24–26].

Despite the progress it has made in TB control, China still has the world’s third largest TB epidemic in 2019 with 833 thousand new TB cases [28]. More than 20% of relapse TB patients who have previously treated in China are multi-drug-resistant TB (MDR-TB), likely due to previously poor treatment [28]. To address this issue, China-Gates Foundation TB Control Program (phase three) introduced and expanded a new comprehensive model of TB control in China since 2017. E-learning for TB health workers was an integral part of that program. In the past two decades, increasing E-learning tools focus on TB training are available, such as The Structure Operational Research and Training (SORT IT) course developed by The International Union Against Tuberculosis and Lung Disease and Médecins Sans Frontières (MSF) [29, 30]. However, few studies have explored the effectiveness of E-learning tools for health care providers. This study aims to evaluate whether E-learning can improve TB health personnel’s knowledge and behaviour, in order to provide policy recommendations for improving the utilization of E-learning in CME.

## Methods

### Intervention design

This E-learning subproject was implemented from May 2017 to June 2019 among the three project provinces (Zhejiang represents the most developed eastern area in China, while Jilin is from the less developed central area and Ningxia represents the least developed western area). In each province, we selected two cities as pilot area for E-learning project according to their level of socioeconomic development and TB health service capacity [number of TB health workers, Gross Domestic Product (GDP) per capita, information technology development].

Two key interventions were designed in the E-learning project [31]. First, the "National TB Telemedicine Consultation and Training Platform" is a live, synchronous training platform developed by Clinical Centre on Tuberculosis (hereinafter referred to as "synchronous training"). This platform focused on complex clinical conditions for TB clinical staff at the county level and above. The platform provides multiple formats of training sessions, including lectures, case studies, and online meetings. Second, “China TB prevention Online Training Website”, an asynchronous training and qualification system (hereinafter referred to as "asynchronous training") developed for all TB health workers, including clinical doctors, public health physicians, and primary care medical staff. The online system focused on basic theory and routine treatment according to the latest version of the national TB treatment and control guideline. The website sessions are provided by recorded videos, which created more flexibility of time and space for health personnel. Both synchronous and asynchronous training sessions were delivered by national level TB experts [32].

### Evaluation design

**Fig. 1:**
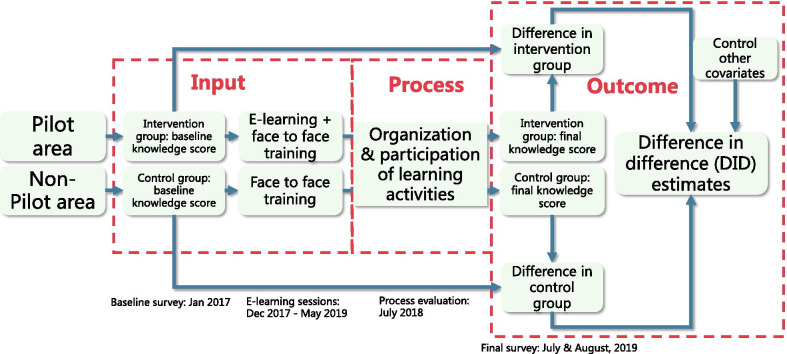
Evaluation design

This evaluation study was a convergent mixed method study. With the conventional “Input-Process-Output” framework, we selected representative study sites from multiple levels (provincial, city, county, township, and village-level). Before the intervention, in both intervention group (pilot area) and control group (non-pilot area), we selected one city in each province as study sites according to their level of socioeconomic development (for example, GDP per capita, type of TB health service delivery model, etc.). Based on similar criteria, two counties from each city and three towns from each county (including at least one remote or mountainous town) were also selected. We also conducted a baseline survey before the intervention to capture the baseline knowledge level of TB health workforces. During the intervention (May 2017–June 2019), we monitored the process of training by monthly reports from the IT system (quantitative data) and the interviews of organizers, lecturers, and participators (qualitative data). These data would help us to open the black box of mechanism of E-learning. After the intervention, we re-examined the knowledge level of TB health workforce and employed the difference in difference (DID) method to capture the effectiveness of E-learning interventions. (Fig. 1).

We conducted three waves of field trips (baseline, process, and the final evaluation) in 2017 (January), 2018 (July and August), and 2019 (July and August), respectively. Two different types of data were collected for analysis. Firstly, we performed two waves of questionnaire surveys among TB health workers during the baseline (2017, pre-training) and final evaluation (2019, post-training), which provided quantitative data about demographic information, training needs, participants' reactions to the training program, and a 10-question TB quiz. The TB quiz were prepared by national experts from the National Clinical Centre on Tuberculosis, Chinese Centre for Disease Control and Prevention (China CDC). Three different types of quizzes are used for clinical, public health physicians, and primary care workers. We also asked several questions on behaviour change in the questionnaires; the participants could choose whether they can put what they have learned into daily practices. All TB health workers on duty on the day of fieldwork were invited to fill in the questionnaire. In total, 555 TB-related health workers completed the baseline questionnaire survey and 757 completed the final questionnaire survey (Table 1, Additional file 1).

**Table 1:** Sample size and general information of TB health worker survey

		Baseline survey			Final survey		
		Clinical physicians	Public health physicians	Primary care workers	Clinical physicians	Public health physicians	Primary care workers
Total		143	79	274	190	92	476
Province	Zhejiang	52	31	54	58	45	132
	Jilin	63	24	129	86	24	164
	Ningxia	28	24	91	46	23	180
Level of institutions	Provincial	15	24	-	54	25	-
	Prefecture	36	30	-	75	33	-
	County	92	25	-	61	34	-
Areas	Non-pilot areas	79	37	165	84	50	160
	Pilot areas	64	42	109	106	42	316
Training participation	Never participated	-	-	-	26	40	337
	Participated in asynchronous activities	-	-	-	134	48	139
	Participated in synchronous activities	-	-	-	143	-	-
Gender	Female	81	46	98	115	61	171
	Male	61	33	176	75	31	301
Age group	< 30	10	8	30	29	16	43
	30–50	113	62	190	145	60	316
	> 50	20	9	50	16	16	117
Education level	Technical school or below	21	4	90	4	6	177
	College (3 years)	30	20	149	31	18	249
	University or above (≥ 5 years)	92	55	33	155	68	46
Professional title	None or primary	37	23	228	76	29	445
	Middle	64	20	37	55	30	23
	Senior	42	26	5	59	32	4

Data source: TB health worker survey. *CDCs* Centres for Disease Control and Prevention

Qualitative data were collected to explore the impacts on behaviour change. With the help of coordinators, participants were also recruited for key informant interviews and focused group discussions (FGDs) at each level with a purposive sampling method. Key informant interviews were conducted with program officers at national and provincial levels, trainers and trainees of E-learning activities. According to the size of the working team in each designated hospital and CDC, FGDs were convened with 6–8 TB-related doctors (clinician group), 2–3 public health physicians (public health physician group), or 1–3 primary care workers (primary care worker group). The topic guides included issues on their motivation to participate, the impact of E-learning on their knowledge and behaviours, and their learning or teaching experience (from both the trainers and trainees). The interviews and FGDs were conducted in a quiet meeting room or office room without any other irrelevant people. A senior researcher conducted the interviews and FGDs as the interviewer or facilitator, with a junior researcher as observer and notetaker. The talks were recorded after participants signed the consent forms. In total, we conducted 30 key informant interviews and 44 FGDs (Additional file 2).

### Data analysis

For both quantitative and qualitative data, analyses were conducted around two dimensions. First, the impact of E-learning on physicians’ knowledge about TB. A comparison of knowledge scores was made before and after the training activities among TB health workers. Besides, we used two different identification strategies to capture the association between knowledge improvement and E-learning interventions. We employed the following DID model to control for the unobserved time-invariant fixed effects and common time-varying trends (formula 1). Difference in difference is a widely-used identification strategy in the area of policy analysis and health service research [33–36]. We take the training intervention as a quasi-experiment in our study. Our sample is broken down into four groups: the control group before the intervention, the control group after the intervention, the treatment group before the intervention, and the treatment group after the intervention.

1 $$Y={\beta }_{0}+{\beta }_{1}t+{\beta }_{2}Training*t+{\beta }_{3}X+{\alpha }_{i}+\varepsilon$$

The subscript* i* indicates the medical institutions (for clinical and public health physicians) or township (for primary care workers). The dependent variables *Y* represents the TB knowledge score among TB health workers.${\alpha }_{i}$ is a series of institutional-level fixed effects (for clinical and public health physicians) or township-level fixed effects (for primary care workers) that controls for the unobserved time-invariant heterogeneity across institutions or towns. ε refers to the error term. *X* is a set of covariates including the health workers’ gender, age, permanent post, professional titles, education level, medical discipline, length of service, and monthly income. The key variable of interest is a dummy variable—*Training*. It equals 1 for physicians in pilot areas after the implementation training program and 0 elsewhere. By pooling independent cross-sectional data across two years (one before the training and one after the training), we could estimate the effect of training with the following DID estimator—${\beta }_{2}$ (formula 2):

2 $$\widehat{{\beta }_{2}}=\left(\overline{{Y }_{t=1,treatment}}-\overline{{Y }_{t=0,treatment}}\right)-\left(\overline{{Y }_{t=1,control}}-\overline{{Y }_{t=0,control}}\right)$$

Multiple linear regression was also conducted to capture which training format is the most effective in terms of knowledge improvement (formula 3), and to quantify the dose–response relationship between training participation and the accumulation of TB knowledge (formula 4). The subscript *f*, *s*, and *a* indicate three different formats of training: face-to-face training, synchronous E-learning, and asynchronous E-learning. There are two variables of interest: *Training* here is a dummy variable; it equals 1 if the physician has participated in a specific type of training. $T\_times$ is a continuous variable that stands for the number of sessions related to this specific type of training. In other words, if there ${\beta }_{2}$, ${\beta }_{3}$, or ${\beta }_{4}$ statistically significant in the following model, we can conjecture that the training intervention may have a positive or negative impact on physicians’ knowledge.

3 $$Y={\beta }_{0}+{\beta }_{1}t+{\beta }_{2}{Training}_{f}+{\beta }_{3}{Training}_{s}+{\beta }_{4}{Training}_{a}+{\beta }_{5}X+{\alpha }_{i}+\varepsilon$$

4 $$Y={\beta }_{0}+{\beta }_{1}t+{\beta }_{2}{T\_times}_{f}+{\beta }_{3}{T\_section}_{s}+{\beta }_{4}{T\_times}_{a}+{\beta }_{5}X+{\alpha }_{i}+\varepsilon$$

The second dimension was the effectiveness of E-learning on physicians’ TB control practice, which is mostly based on qualitative data with quantitative supplementary data. We transcript and analysed the interviews using a hybrid approach in thematic analysis. The analytical framework was developed based on both the topic guides (inductive) and emerging issues (deductive) from the interviews and FGDs [37]. The quantitative data were analysed using Stata 14.0 (StataCorp, College Station, TX, USA) and the qualitative data were analysed in MAXQDA 2018 (VERBI GmbH, Berlin, German).

## Results

### Effectiveness of E-learning on TB health workers’ knowledge

After the E-learning training, the knowledge level of TB health workers has been significantly improved. The average score of the clinical quiz raised from 65 to 82 points (*P* < 0.001), the average score of the public health quiz raised from 65 to 71 points (*P* = 0.009), and the average score of the primary care quiz raised from 79 to 85 points (*P* < 0.001).

However, the further univariate analysis suggested that the change score among the clinical physicians in the pilot area (+ 17 points) was not statistically different from the physicians in the non-pilot area (+ 18 points, *P* = 0.714). Similar results were observed in public health physicians (pilot area: + 4 points, non-pilot area: + 9 points, *P* = 0.772). Despite the null effect among health workers from the county-level and above, the change score of primary care personnel in pilot area (10 points) was statistically higher than that in non-pilot area (4 points, *P* < 0.001, Table 2).

**Table 2:** Descriptive statistics of TB knowledge change of pre-and post-training for TB health workers (mean ± standard deviation, full marks = 100)

		Baseline knowledge score			Final knowledge score		
		Clinical physicians	Public health physicians	Primary care workers	Clinical physicians	Public health physicians	Primary care workers
Total		65 ± 21	65 ± 16	79 ± 19	82 ± 19***	71 ± 14***	85 ± 16***
Intervention	Non-pilot areas	66 ± 22	63 ± 19	82 ± 18	84 ± 17***	72 ± 14*	86 ± 14
	Pilot areas	63 ± 20	66 ± 13	74 ± 19	80 ± 20***	70 ± 15*	84 ± 17***
Training participation	Never participated	-	-	-	81 ± 20	68 ± 18	83 ± 17
	Participated in asynchronous activities	-	-	-	83 ± 19	73 ± 11	90 ± 13
	Participated in synchronous activities	-	-	-	83 ± 18	-	-

Data source: TB health worker survey. *CDCs* Centres for Disease Control and Prevention

***P < 0.01, **P < 0.05, *P < 0.1 for pre-post difference

The DID model showed that after controlling for institutional fixed effects, personal characteristics and other control variables, the improvement of the knowledge level of pilot area is slightly lower than that of non-pilot area for both clinical physicians and public health physicians, and the difference is not significant. However, the results of primary care workers shown a different pattern: the improvement of knowledge level among primary care workers in the pilot area was significantly higher than that in the non-pilot area, with an average improvement of 8.0 points (*P* < 0.001). On the contrary, the knowledge level of primary care workers in all sample areas did not change significantly before and after the intervention (Table 3).

**Table 3:** Effect of China-Gates TB training program on health workers’ knowledge: difference-in-difference model

Independent variables	Dependent variables: knowledge score		
	(1) Clinical physicians	(2) Public health physicians	(3) Primary care workers
Time (Final = 1; Baseline = 0)	17.26***	8.88**	1.75
	(3.09)	(4.34)	(1.41)
Time × Area (pilot area = 1, non-pilot area = 0)	− 2.16	− 4.09	7.97***
	(3.82)	(6.00)	(2.22)
Controls for institution fixed effect and individual characteristics	Yes	Yes	Yes
Sample size	334	168	721
Adjusted R^2^	0.364	0.558	0.365

Data source: TB health worker survey. Standard deviation in parentheses. Control variables including dummy variables (township-level or institutional fixed effect, gender, permanent post, academic major, professional titles, and job positions) and continuous variables (age, monthly income, years of medical education, and length of service)

TB: Tuberculosis

***P < 0.01, **P < 0.05, *P < 0.1

The results of multiple linear regression showed that for the clinical physicians who actually participated in the synchronous E-learning sessions, their clinical knowledge has improved, compared to their colleagues who did not participate. After controlling for institutional fixed effects and personal characteristics, the TB knowledge level of clinical physicians who have participated in synchronous learning activities increased by 7.3 points on average (*P* = 0.023). For each time they participate in a synchronous session, their clinical TB quiz score could increase by 0.3 points on average (*P* = 0.034). Asynchronous learning activities have no significant impact on the knowledge level of clinical and public health physicians, but a significant positive effect was seen among primary care staff. Compared with those who did not participate, trained primary care workers had a higher average score of 10.9 points (*P* < 0.001). For each module they attended, their knowledge score could increase 1.8 points on average (*P* < 0.001). Moreover, after they obtain a certificate (indicates that all modules are finished), the average score of the participants could increase by 11.2 points (*P* < 0.001). Traditional face-to-face training has no significant impact on the knowledge improvement for all medical staff. We didn’t find any statistically significant improvement for the public health physicians in terms of their TB knowledge level, no matter what format of training they have taken (Table 4).

**Table 4:** Effect of China-Gates TB training program on health workers’ knowledge: multiple linear regression model

Independent variables	Dependent variables: knowledge score					
	(1) Clinical physicians	(2) Public health physicians	(3) Primary care workers	(4) Clinical physicians	(5) Public health physicians	(6) Primary care workers
Time (Final = 1; Baseline = 0)	12.38***	6.12*	0.64	12.57***	7.57**	1.75
	(3.38)	(3.96)	(1.35)	(2.99)	(3.79)	(1.41)
Participated in the face-to-face training (Yes = 1, No = 0)	1.63	2.94	0.40	-	-	-
	(2.97)	(5.08)	(2.20)	-	-	-
Participated in the synchronous learning activities (Yes = 1, No = 0)	7.28**	-	-	-	-	-
	(3.19)	-	-	-	-	-
Participated in the asynchronous learning activities (Yes = 1, No = 0)	1.63	2.94	10.94***	-	-	-
	(2.97)	(5.08)	(1.92)	-	-	-
Count of face-to-face sessions	-	-	-	− 0.45**	− 0.13	0.0004
	-	-	-	(0.20)	(0.68)	(0.18)
Count of synchronous activities	-	-	-	0.34**	-	-
	-	-	-	(0.16)	-	-
Count of asynchronous activities	-	-	-	0.40	− 0.15	1.84***
	-	-	-	(0.41)	(1.13)	(0.31)
Obtained the certificate (Yes = 1, No = 0)	-	-	-	0.75	0.51	11.24***
	-	-	-	(3.55)	(7.92)	(2.08)
Controls for institution fixed effect and individual characteristics	Yes	Yes	Yes	Yes	Yes	Yes
Sample size	333	169	721	332	169	719
Adjusted R^2^	0.357	0.546	0.382	0.376	0.539	0.392

Data source: TB health worker survey. Standard deviation in parentheses. Control variables including dummy variables (township-level or institutional fixed effect, gender, permanent post, academic major, professional titles, job positions) and continuous variables (age, monthly income, years of medical education, length of service)

*TB* Tuberculosis

***P < 0.01, **P < 0.05, *P < 0.1

We conducted three different types of sensitivity analyses. For the multiple linear regression model, the dependent variable was replaced from the original score to the Z-score ($Z=\frac{x-\mu }{\sigma }$, $x$ is the raw score,$\mu$ is the mean and $\sigma$ is the standard deviation) and the results did not change significantly. For the DID model, two placebo tests were conducted (test 1: rerandomization; test 2: re-allocation according to the quality control subproject—another subproject of China-Gates TB control program). After regrouping, the interaction coefficients between the pilot area and the time variable were no longer significant, indicating that the intervention effects we observed were not due to a random factor or the effects of other pilot projects. In the leave-one-out analysis, we run the DID model again by excluding one of the 13 counties each time. Although the interaction terms were only significant at the α = 0.1 level after excluding the samples of Tongxin or Nong’an counties, they were still significantly positive at the α = 0.05 level after excluding the other 11 counties one by one, indicating that the improvement in the level of knowledge of the medical staff was significant and robust (Additional file 3).

### Effectiveness of E-learning on healthcare worker’s behaviour

The E-learning project not only improved the knowledge of TB among medical staff, but also enabled some medical staff to apply the knowledge they learned in their daily practice, which improved their quality of care. We measured their perceptions of the impact that E-learning would have on their practice as a proxy indicator of behaviour. Overall, there was a high heterogeneity: behaviour change varied among medical staff from different regions and at different levels of institutions.

According to the results of the questionnaire survey, 56.0% (93/166) of the 166 synchronous E-learning session participants agreed that “the training knowledge can be applied to my work”. The univariate analysis suggested that medical staff at the provincial level (*P* < 0.001), with bachelor degree or above (*P* < 0.001), and from pilot area (*P* = 0.039) were more likely to agree with this argument. For asynchronous E-learning training, 57.3% of the 309 participants have expressed a similar view (177/309). Compared with the results of synchronous E-learning, a higher proportion of county-level health workers or those with low-level of education agreed on that argument in asynchronous E-learning training (Table 5).

**Table 5:** Percentage of TB health workers felt the knowledge and skills they learned through E-learning program can be applied in their daily practices

		Clinical and Public health physicians		Primary care workers (*n* = 136)
		Synchronous E-learning activities (*n* = 166)	Asynchronous E-learning activities (*n* = 173)	
Total		56.0	57.2	57.4
Province	Zhejiang	67.4	61.9	84.2
	Jilin	54.8	56.0	53.3**
	Ningxia	45.5	51.4	50.0**
Level of institutions	Provincial	76.7	72.9	-
	Prefecture	50.8***	59.7***	-
	County	34.9***	41.4***	-
Areas	Non-pilot areas	44.2	49.3*	56.5
	Pilot areas	61.4	63.0	57.8
Education level†	Technical school or below	28.0	44.1	48.2
	College (3 years)	61.1***	61.1	61.0
	University or above (≥ 5 years)	60.7***	58.1	70.0

Data source: TB health worker survey. *TB* Tuberculosis

***P < 0.01, **P < 0.05, *P < 0.1 for Chi-Squared Test

In the interview, medical staff who believed the training could change their practice behaviours mentioned that the knowledge they learned in the China-Gates E-learning helped them in three ways: First, doctors at the city and county-level institutions or junior doctors learned what they did not know before in E-learning sessions, such as standardized TB diagnosis and treatment, or they got a more clear and deeper understanding about these knowledges. They can adopt what they have learned when they encounter the same problem in their practices. The second is for doctors at the provincial and prefecture-level institutions, they get an opportunity to see more intractable cases through training, and learn cutting-edge knowledge of TB diagnosis and treatment from national experts. Both of them were helpful to cultivate their clinical thinking, which made them better prepared for complex clinical problems. The third is that the training itself creates a positive learning environment in the department and causes imperceptible improvements in doctor’s study habit.

The first time I listened to some sessions about immunization, molecular biology, and genes, I was also confused, but after listening several times, I have come gradually to understand what they are talking about. I guess it is just what we called “gradual progression”, we are cultivating a learning habit by training, and then we can make progress in our clinical (practice). (Provincial TB health professional, FGD in Jilin).

During the interview, three main reasons were also put forward by the medical staff who thought they could not apply the knowledge into their practices. First of all, some physicians complained that they have not remembered the new knowledge, or the topics are too easy for them, either condition would offer any help to their practice. The second reason is due to the hardware equipment. Some county-level medical personnel mentioned that even if they have improved their knowledge by training, the equipment, available drugs, and laboratory capabilities of their medical institutions cannot support them to change—they couldn’t do the same lab test and treatment as national hospitals usually do. The third reason is that the knowledge learned was not relevant to their daily work. Many public health physicians in the interviews reported that the training contents they received did not cover the topics in their daily work and were not helpful.

The problems in daily work are not reflected in the training. I don't understand the problems in the statistical report, but the training has nothing about this. (County TB public health physician’s interview in Ningxia).

## Discussion

Considering the lack of high-quality educational resources for grassroots doctors in LMICs, and the great challenges that traditional continuing medical education faces due to the COVID-19 pandemic, E-learning CME has important policy significance in LMICs. Our research results have demonstrated that E-learning can significantly improve the knowledge level of TB health workers. Moreover, it has a greater effect on primary care workers, and plays an important role in "equalization of basic theory, knowledge, and essential skills” among different level of institutions. However, we didn’t see the obvious benefits for public health physicians.

Our results are quite different from most previous studies in higher education settings, most of which identified negative effects of online courses on college student education [38–52]. These differences illustrate that the impact of modern educational technology on education is complex. It may improve students’ access to high-quality resources and therefore have a positive impact on students’ learning outcomes, and it may also affect the learning experience and have a negative impact. Most of the existing research focuses on comparing face-to-face and online training in which the students have access to the same educational resources. In this condition, the online training format may prohibit students’ learning participation, resulting in negative learning outcomes. Few studies have explored the effect of E-learning in the context of primary care in LMICs, who had no access to national and provincial medical education resources without E-learning opportunities.

Our results indicate different benefits based on the training format and the target audiences. First, according to the target audience, primary health workers benefited the most from training, while public health personnel benefited the least. Mismatch of the supply & demand for training and environmental factors are the main reasons for the differences [31]. In the China-Gates TB control project, the training supply and demand for clinical physicians and primary care personnel are well matched, while the training contents for public health physicians were not consistent with what they demanded. Secondly, in terms of training format, this study found that these two types of E-learning training (synchronous and asynchronous) were both effective in some ways, but the face-to-face training was not significantly effective. This is consistent with the relevant research results in other fields of medical continuing education [53], suggesting that the existing traditional CME model needs to be reformed.

This study has several limitations. Firstly, we did not collect panel data at the individual level. Due to data availability, the regression analysis may have endogenous missing variables. Therefore, we employed three different strategies in statistical analysis to enhance the validity of the results. Secondly, this study did not involve the objective measurement of physicians' behaviour to evaluate the effect of training intervention. Instead, we used the self-reported behavioural change after the training. Existing evidence in LMICs has shown that training may not change the providers prescribing behaviours even after they learned the guideline [54, 55], so our results need to be interpreted with caution. Third, the whole China-Gates TB project is a set of comprehensive and complex interventions. Many project counties have multiple interventions at the same time, such as the development of new information systems, application of electronic medical monitors, and the health insurance payment reform for tuberculosis patients. Given the study design chosen, it is difficult to distinguish the specific effects of each intervention. However, sensitivity analysis showed no evidence that other interventions have a direct impact on the knowledge level of medical staff. Fourth, we didn’t conduct any follow-up survey after 2019, so we don’t know whether E-learning would produce lasting increases in knowledge scores for health care providers.

## Conclusions

This study indicates different impact of E-learning based on the training format and the target audience. For TB clinical and primary care health workers, E-learning interventions are associated with a higher TB knowledge level. However, E-learning activities didn’t provide significant benefits for public health physicians. Traditional face-to-face training has no significant impact on all types of medical staff. Future studies on E-learning activities in CME should aim to create the learning environment within the health system to realize the full potentials of E-learning. Future research should also further explore the effect of E-learning on physician’s behavioural practice, like prescribing behaviours.

## Supplementary Information

**Additional file 1. **Baseline characteristics for intervention and control group (2017).

**Additional file 2. **Sample size for key informant interviews and FGDs.

**Additional file 3. **Sensitivity analysis for multiple linear regression model.

## Data Availability

The datasets used and/or analysed during the current study are available from the corresponding author on reasonable request.

## Abbreviations

TB: Tuberculosis
CME: Continuing medical education
DID: Difference-in-difference
COVID-19: Coronavirus disease 2019
GDP: Gross domestic product
CDC: Centre for Disease Control and Prevention
FGDs: Focused group discussions
