# Supplementary material for: The effectiveness of E-learning in continuing medical education for tuberculosis health workers: a quasi-experiment from China
Source: Infect Dis Poverty. 2021 May 18;10:72. doi: 10.1186/s40249-021-00855-y (PMC8129609; doi:10.1186/s40249-021-00855-y)
Supplement: Supplementary file 1 — Additional file 1. Baseline characteristics for intervention and control group (2017). [file 40249_2021_855_MOESM1_ESM.docx]

**Appendix 1: baseline** **characteristics for intervention and control group (2017)**

**Baseline** **characteristics for intervention and control group: health workers from the county-level and above**

|  | Intervention group | Control group | P-value |
| --- | --- | --- | --- |
| Number of institutions | 13 | 23 | - |
| Level of institutions: prefecture-level or above (no. %) | 5 (38.46%) | 7 (30.43%) | 0.143 |
| Number of TB health workers in the institution (Mean ± SD) | 10.56±2.78 | 6.83±0.89 | 0.104 |
| Age (in years, mean ± SD) | 42.45±0.97 | 40.79±0.71 | 0.150 |
| Sex: male (%) | 37.23 | 37.97 | 0.827 |
| Education: university or above (%) | 43.16 | 59.49 | 0.046 |
| Lengths of service (in years, mean ± SD) | 20.53±1.00 | 19.35±0.81 | 0.337 |
| Staff types: doctors (%) | 35.11 | 33.12 | 0.489 |
| Professional title: senior or above (%) | 27.37 | 29.75 | 0.596 |
| Monthly income (in RMB, mean ± SD) | 7414±1540 | 4850±151 | <0.001 |

**Baseline** **characteristics for intervention and control group: primary care providers**

|  | Intervention group | Control group | P-value |
| --- | --- | --- | --- |
| Number of health workers | 108 | 164 | - |
| Level of institutions: primary care centres or township hospitals (no. %) | 13 (12.04%) | 24 (14.63%) | 0.541 |
| Age (in years, mean ± SD) | 44.94±0.71 | 39.95±0.82 | 0.002 |
| Sex: male (%) | 61.98 | 56.97 | 0.006 |
| Education: collage and above (%) | 57.94 | 72.73 | 0.011 |
| Lengths of service (in years, mean ± SD) | 22.23±0.83 | 17.73±0.92 | 0.023 |
| Monthly income (in RMB, mean ± SD) | 3332±372 | 2304±94 | 0.016 |
| Professional title: senior or above (%) | 27.00 | 9.09 | 0.001 |

Data source: TB health worker survey. SD: Standard deviation. RMB: Renminbi Yuan.
