# Supplementary material for: The effectiveness of E-learning in continuing medical education for tuberculosis health workers: a quasi-experiment from China
Source: Infect Dis Poverty. 2021 May 18;10:72. doi: 10.1186/s40249-021-00855-y (PMC8129609; doi:10.1186/s40249-021-00855-y)
Supplement: Supplementary file 2 — Additional file 2. Sample size for key informant interviews and FGDs. [file 40249_2021_855_MOESM2_ESM.docx]

**Appendix 2: Sample size for key informant interviews and FGDs**

|  | **Project management personnel** | **Trainers** | **Doctors** | **Public health physicians** | **Primary care workers** |
| --- | --- | --- | --- | --- | --- |
| **Key informant interviews (number of participants, total N = 30)** | | | | | |
| **National level: China CDC** | NA | 2 | 2 | NA | NA |
| **Ningxia: Yinchuan (Capital)** | 4 | 1 | NA | 4 | NA |
| **Jilin: Changchun Capital** | 4 | 3 | NA | 2 | NA |
| **Zhejiang: Hangzhou (Capital)** | 5 | 1 | NA | 2 | NA |
| **FGDs (number of groups, total N = 44)** | | | | | |
| **National level: China CDC** | 1 | NA | NA | NA | NA |
| **Ningxia: Yinchuan (Capital)** | NA | NA | 2 | NA | NA |
| **Ningxia: City-level** | NA | NA | 3 | 2 | NA |
| **Ningxia: County-level** | NA | NA | 3 | 2 | 3 |
| **Jilin: Changchun (Capital)** | NA | NA | 2 | NA | NA |
| **Jilin: City-level** | NA | NA | 3 | 1 | NA |
| **Jilin: County-level** | NA | NA | 3 | 1 | 3 |
| **Zhejiang: Hangzhou (Capital)** | NA | NA | 2 | NA | NA |
| **Zhejiang: City-level** | NA | NA | 3 | 2 | NA |
| **Zhejiang: County-level** | NA | NA | 3 | 2 | 3 |

NA: Not available.
