# Supplementary material for: The effectiveness of E-learning in continuing medical education for tuberculosis health workers: a quasi-experiment from China
Source: Infect Dis Poverty. 2021 May 18;10:72. doi: 10.1186/s40249-021-00855-y (PMC8129609; doi:10.1186/s40249-021-00855-y)
Supplement: Supplementary file 3 — Additional file 3. Sensitivity analysis for multiple linear regression model. [file 40249_2021_855_MOESM3_ESM.docx]

**Appendix 3: Sensitivity analysis**

**Sensitivity analysis for multiple linear regression model**

| **Independent variables** | **Dependent variables: Z-score** | | | | | |
| --- | --- | --- | --- | --- | --- | --- |
|  | **(ⅰ)**  **Clinical physicians** | **(ⅱ)**  **Public health physicians** | **(ⅲ)**  **Primary care workers** | **(ⅳ)**  **Clinical physicians** | **(ⅴ)**  **Public health physicians** | **(ⅵ)**  **Primary care workers** |
| Time (Final=1；Baseline=0) | 0.695*** | 0.240 | 0.037 | 0.548*** | 0.297** | 0.065 |
|  | (0.154) | (0.155) | (0.079) | (0.130) | (0.149) | (0.074) |
| Participated in the face-to-face training (Yes=1, No=0) | 0.256* | 0.100 | 0.023 | — | — | — |
|  | (0.148) | (0.190) | (0.128) | — | — | — |
| Participated in the synchronous learning activities (Yes=1, No=0) | 0.258** | — | — | — | — | — |
|  | (0.130) | — | — | — | — | — |
| Participated in the asynchronous learning activities (Yes=1, No=0) | -0.043 | 0.115 | 0.636*** | — | — | — |
|  | (0.136) | (0.200) | (0.112) | — | — | — |
| Count of face-to-face sessions | — | — | — | -0.020** | -0.003 | 0.001 |
|  | — | — | — | (0.009) | (0.026) | (0.011) |
| Count of synchronous activities | — | — | — | 0.015** | — | — |
|  | — | — | — | (0.007) | — | — |
| Count of asynchronous activities | — | — | — | 0.017 | -0.005 | 0.107*** |
|  | — | — | — | (0.018) | (0.442) | (0.018) |
| Obtain the certificate (Yes=1, No=0) | — | — | — | 0.033 | 0.020 | 0.654*** |
|  | — | — | — | (0.155) | (0.311) | (0.121) |
| Controls for institution fixed effect and individual characteristics | Yes | Yes | Yes | Yes | Yes | Yes |
| Sample size | 333 | 169 | 721 | 332 | 169 | 719 |
| Adjusted R^2^ | 0.357 | 0.546 | 0.382 | 0.376 | 0.539 | 0.392 |

Data source: TB health worker survey. Standard deviation in parentheses. Control variables including dummy variables (township-level fixed effect, type of institutions, gender, “Bianzhi”, education level, academic major, professional titles) and continuous variables (age, monthly income).

*** *P* < 0.01, ** *P* < 0.05, * *P* < 0.1.

**Sensitivity analysis for Difference-in-Difference model: Placebo test**

| **Independent variables** | **Dependent variables: raw score** | | |
| --- | --- | --- | --- |
|  | **Original model** | **Test 1: rerandomization †** | **Test 2: re-allocation according to the quality control subproject ‡** |
| Time (Final=1；Baseline=0) | 1.753 | 3.597*** | 4.442*** |
|  | (1.414) | (1.361) | (1.473) |
| Time×Pilot areas (Pilot areas=1; Nonpilot areas=0) | 7.965*** | 3.651 | 0.601 |
|  | (2.216) | (2.359) | (2.172) |
| Controls for township-level fixed effect, institutional and individual characteristics | Yes | Yes | Yes |
| Constant | 97.120*** | 94.338*** | 96.191*** |
|  | (3.815) | (5.281) | (5.180) |
| Sample size | 721 | 721 | 721 |
| Adjusted R^2^ | 0.365 | 0.356 | 0.354 |

Data source: TB health worker survey. Standard deviation in parentheses. Control variables including dummy variables (township-level fixed effect, type of institutions, gender, “Bianzhi”, education level, academic major, professional titles) and continuous variables (age, monthly income).

*** *P* < 0.01, ** *P* < 0.05, * *P* < 0.1.

† Re-allocation result for the test 1: Pilot areas – Jiangshan, Longyou, Taonan, Qingtongxia; Nonpilot areas – Tongxiang, Changshan, Zhuji, Nong’an, Dehui, Zhenlai, Zhongwei, Haiyuan, Tongxin.

‡ Re-allocation result for the test 2: Pilot areas – Nong’an, Dehui, Tongxin, Qingtongxia; Nonpilot areas – Jiashan, Tongxiang, Changshan, Longyou, Zhuji, Taonan, Zhenlai, Zhongwei, Haiyuan.

**Sensitivity analysis for Difference-in-Difference model: Leave-1-out analyses**

| **County excluded in the analysis** | **Dependent variables: raw score** | | |
| --- | --- | --- | --- |
|  | Time×Pilot areas (DID interaction term) | Sample size | Adjusted R^2^ |
| Original model | 7.965*** | 721 | 0.365 |
|  | (2.216) |  |  |
| Jiashan | 7.634*** | 677 | 0.361 |
|  | (2.287) |  |  |
| Tongxiang | 7.147*** | 670 | 0.325 |
|  | (2.352) |  |  |
| Changshan | 8.417*** | 703 | 0.373 |
|  | (2.229) |  |  |
| Longyou | 8.951*** | 664 | 0.349 |
|  | (2.299) |  |  |
| Zhuji | 7.934*** | 711 | 0.361 |
|  | (2.228) |  |  |
| Nong’an | 3.383* | 645 | 0.363 |
|  | (2.536) |  |  |
| Dehui | 5.110** | 656 | 0.387 |
|  | (2.363) |  |  |
| Taonan | 10.655*** | 655 | 0.392 |
|  | (2.263) |  |  |
| Zhenlai | 9.021*** | 648 | 0.348 |
|  | (2.241) |  |  |
| Zhongning | 7.795*** | 671 | 0.363 |
|  | (2.403) |  |  |
| Haiyuan | 15.190*** | 663 | 0.431 |
|  | (2.305) |  |  |
| Tongxin | 3.925* | 624 | 0.375 |
|  | (2.288) |  |  |
| Qingtongxia | 7.334*** | 665 | 0.332 |
|  | (2.341) |  |  |

Data source: TB health worker survey. Standard deviation in parentheses. Control variables including dummy variables (township-level fixed effect, type of institutions, gender, “Bianzhi”, education level, academic major, professional titles) and continuous variables (age, monthly income).

*** *P* < 0.01, ** *P* < 0.05, * *P* < 0.1.
